# Natural hybridization among three *Rhododendron* species (Ericaceae) revealed by morphological and genomic evidence

**DOI:** 10.1186/s12870-021-03312-y

**Published:** 2021-11-11

**Authors:** Wei Zheng, Li-Jun Yan, Kevin S. Burgess, Ya-Huang Luo, Jia-Yun Zou, Han-Tao Qin, Ji-Hua Wang, Lian-Ming Gao

**Affiliations:** 1 CAS Key Laboratory for Plant Diversity and Biogeography of East Asia, Kunming Institute of Botany, Chinese Academy of Sciences, 650201 Kunming, Yunnan China; 2 University of Chinese Academy of Sciences, 10049 Beijing, China; 3 College of Vocational and Technical Education, Yunnan Normal University, 650092 Kunming, Yunnan China; 4 Department of Biology, Columbus State University, University System of Georgia, 31907-5645 Columbus, GA USA; 5 The Flower Research Institute, Yunnan Academy of Agricultural Sciences, 650205 Kunming, China; 6 Lijiang Forest Biodiversity National Observation and Research Station, Kunming Institute of Botany, Chinese Academy of Sciences, 674100 Lijiang, Yunnan, China

**Keywords:** ddRAD sequencing, Hybrid zone, Morphological trait, Genetic structure, Natural hybridization, *Rhododendron*

## Abstract

**Background:**

Natural hybridization can influence the adaptive response to selection and accelerate species diversification. Understanding the composition and structure of hybrid zones may elucidate patterns of hybridization processes that are important to the formation and maintenance of species, especially for taxa that have experienced rapidly adaptive radiation. Here, we used morphological traits, ddRAD-seq and plastid DNA sequence data to investigate the structure of a *Rhododendron* hybrid zone and uncover the hybridization patterns among three sympatric and closely related species.

**Results:**

Our results show that the hybrid zone is complex, where bi-directional hybridization takes place among the three sympatric parental species: *R. spinuliferum*, *R. scabrifolium*, and *R. spiciferum*. Hybrids between *R. spinuliferum* and *R. spiciferum* (*R.* ×*duclouxii*) comprise multiple hybrid classes and a high proportion of F_1_ generation hybrids, while a novel hybrid taxon between *R. spinuliferum* and *R. scabrifolium* dominated the F_2_ generation, but no backcross individuals were detected. The hybrid zone showed basically coincident patterns of population structure between genomic and morphological data.

**Conclusions:**

Natural hybridization exists among the three *Rhododendron* species in the hybrid zone, although patterns of hybrid formation vary between hybrid taxa, which may result in different evolutionary outcomes. This study represents a unique opportunity to dissect the ecological and evolutionary mechanisms associated with adaptive radiation of *Rhododendron* species in a biodiversity hotspot.

**Supplementary Information:**

The online version contains supplementary material available at 10.1186/s12870-021-03312-y.

## Background

Natural hybridization is frequent in plants and plays a crucial role in the formation and maintenance of species. In recent decades, natural hybridization has been well-documented for numerous herbaceous (e.g., *Helianthus* [1, 2], *Iris* [3], *Senecio* [4], *Viola* [5], *Gagea* [6], *Brassica* [7], *Mimulus* [8]) and some woody plant taxa (e.g., *Pinus* [9, 10], *Ostryopsis* [11, 12], *Rhododendron* [13–16]). For the most part, studies reveal that when two or more closely related species are in sympatry, hybridization frequently occurs and natural hybrid zones are likely to form (e.g. [15, 17, 18]).

Hybrid zone formation is ultimately a response to selection and dispersal mechanisms acting on hybrids and parental species, where parental genomes are combined and functionally selected [19]. In general, there are three main types of hybrid zones that have been documented in plant taxa; these include: tension zones, bounded hybrid superiority zones and mosaic hybrid zones [20, 21]. Within tension zones, hybrids are continually formed, but are selected against due to their low fitness relative to parental species (e.g., *Populus fremontii* × *P. angustifolia* [22, 23]; *Senecio chrysanthemifolius* × *S. aethnensis* [4, 24]). In bounded hybrid superiority zones, hybrids show higher fitness than both parental species in intermediate habitats, but lower fitness in parental habitats (e.g., *Picea glauca* × *P. engelmannii* [25]; *Artemisia tridentata* ssp. *tridentata* × *A. tridentata* ssp. *vaseyana* [26–28]). For mosaic hybrid zones, hybrids have higher fitness than parental taxa across a patchwork of multiple local habitats where the parental species overlap in range (e.g., *Aquilegia formosa* × *A. pubescens* [29]; *Senecio ovatus* × *S. hercynicus* [30]). Ultimately, the evolutionary outcome of natural hybridization between plant taxa is likely to depend on the type of hybrid zone that is formed, where understanding the genetic composition and structure of hybrid zones is an important first step in revealing the evolutionary mechanisms (e.g., selection, dispersal, reproductive isolation) and outcomes associated with natural plant hybridization [17, 31–33].

The genetic composition and structure of plant hybrid zones is often complex (i.e., multiple generations of filial and backcross hybrids), and depending on the strength of pre- and post-reproductive isolation among the parental taxa and their hybrids, hybrid taxa are able to persist, and can even form new species [34, 35]. In many cases, however, the formation and persistence of hybrids depends on the direction and symmetry of hybridization among parental taxa. For example, numerous studies have detected hybridization patterns among parental taxa that are asymmetric [8, 17, 36–38]. In such a case, one likely outcome is asymmetric introgression, where the genome of one parental taxon can become incorporated into the genetic background of another, due in part, to the preferential backcross of hybrids and the numerical asymmetries associated with parental taxa and their hybrids. The detection of such patterns can have profound implications for understanding the formation and persistence of potential hybrid species within a hybrid zone; hybrids, and even some parental taxa, that are not reproductively isolated, are unlikely to persist under such a scenario [39–42]. Alternatively, adaptive introgression can play a central role in the formation and persistence of novel hybrid taxa, and may even lead to the formation of new species. Under this scenario, habitat selection can favor hybrids that incorporate parental genes, which enable them to colonize and persist in novel habitats associated with the hybrid zone (i.e., hybridized habitats, which may be due, in part, to human disturbance [43]). Here, the adaptive introgression of parental traits in hybrids can buffer the deleterious effects of asymmetrical introgression, resulting in the formation and persistence of a complex hybrid zone that is composed of both filial and backcross generations [41, 44–46]. Detection of these unique gene × environment interactions (i.e., the composition of hybrids), in addition to an assessment of hybrid zone structure, may reflect plausible pathways for the formation and persistence of novel hybrid species [21, 40, 47].

*Rhododendron* L. (Ericaceae) is a large and taxonomically complex genus of woody plants that includes more than one thousand species, where more than half of which are endemic to China [48–50], and many species are of ornamental value. The species diversity of *Rhododendron* is rich in the Mountains of Southwest China, which is a global biodiversity hotspot [51, 52]. The morphology of *Rhododendron* species varies considerably, due in part, to substantial adaptive radiation and reticulate evolution of species within the genus [53]. In addition, natural hybridization occurs frequent within *Rhododendron* [13, 16, 54–57]. Previous studies have verified that *R.* ×*duclouxii* H. Lévl., a hybrid taxon between *R. spiciferum* Franch. and *R. spinuliferum* Franch. that has intermediate leaf and flower morphology [54], exists in at least 15 natural hybrid zones in Yunnan, southwest China [16, 58]. A third species, *R. scabrifolium* Franch., also exists within some of these hybrid zones and it remains unknown as to the extent that this species contributes to hybrid formation and hybrid zone structure. It’s worth noting that all three species, in addition to *R.* ×*duclouxii*, are diploid and that *R. scabrifolium* and *R. spinuliferum* are sister taxa [58].

**Fig. 1:**
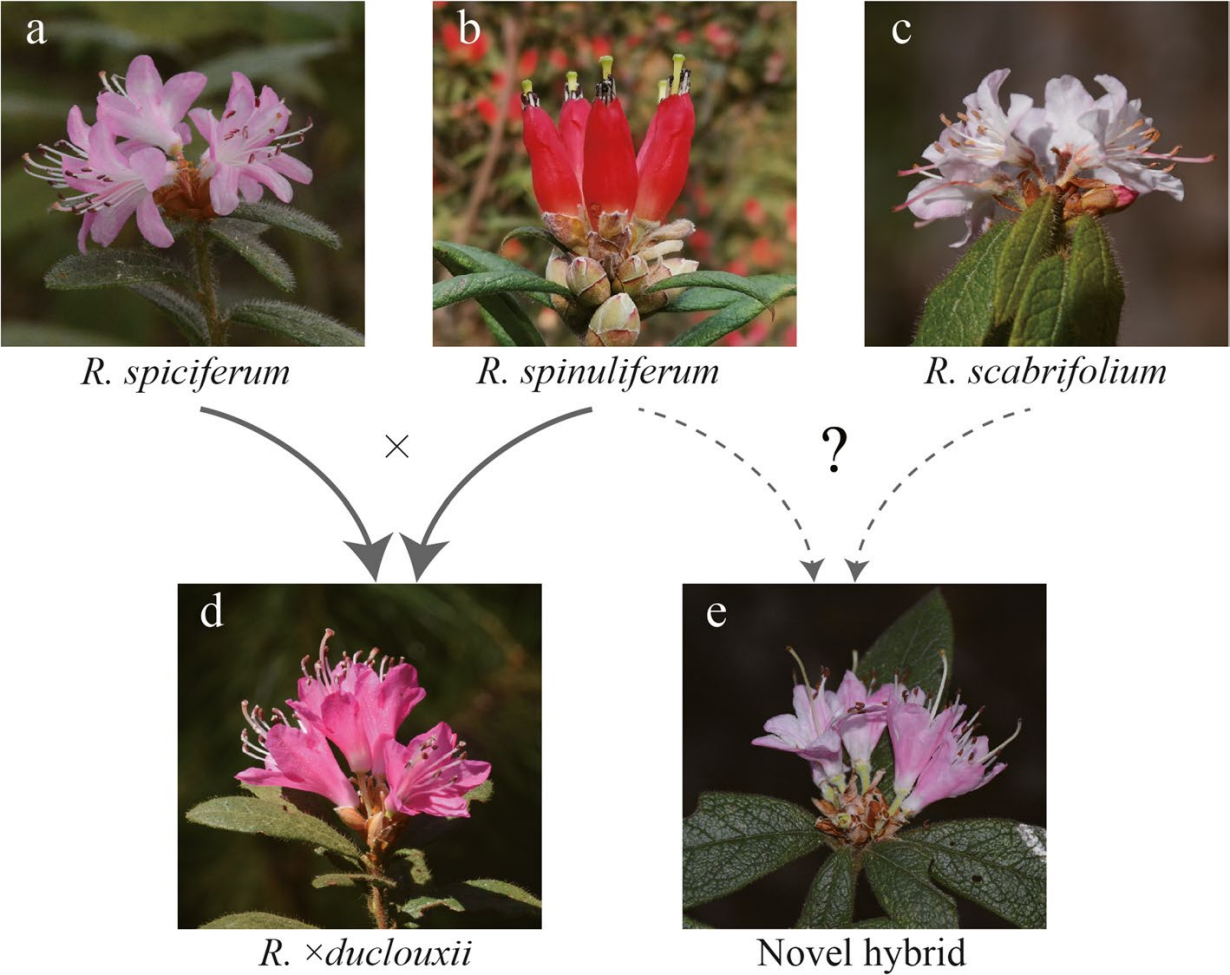
Images of the three parental species of *Rhododendron* and two types of natural hybrids. (a) *Rhododendron spiciferum*; (b) *R. spinuliferum*; (c) *R. scabrifolium* (d) *R. ×duclouxii*; (e) the putative novel hybrid taxon. Solid-line indicates a confirmed result while dashed-line represents our hypothesis for the origin of the novel hybrid taxon

**Fig. 2:**
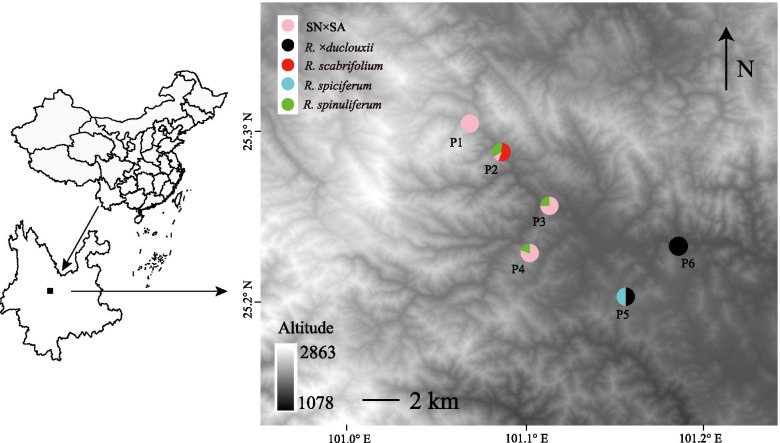
The distribution of the sampled five taxa of *Rhododendron*. Three parent species (*R. spiciferum*, *R. spinuliferum*, *R. scabrifolium*) and two types of hybrids (*R. ×duclouxii*, and the novel hybrid [SN×SA: *R. spinuliferum* × *R. scabrifolium*]) were found in a natural hybrid zone in Yunnan province, China. Samples were collected from six plots within the hybrid zone in this study. The map of China (left) was drawn by R package *maptools* and *ggplot2* and the base map of the plots distribution (right) was download from https://gdex.cr.usgs.gov/gdex/

Recently, a putative novel hybrid taxon is suspected to exist in a region where *R. spiciferum, R. spinuliferum* and *R. scabrifolium* have overlapping ranges. These novel hybrids manifest a suite of morphological traits that not only differ from the three parental taxa at the site, but also differ from the known hybrid taxon *R.* ×*duclouxii* (e.g., flower size, flower color, corolla shape; Fig. 1) and may represent a new hybrid taxon. Here, we use genomic (ddRAD-seq) and morphological data to assess the genetic composition and structure of this *Rhododendron* hybrid zone in Yunnan Province, SW China (Fig. 2). We aim to address the following questions: (1) What is the origin of the novel hybrid taxon? (2) What is the genetic composition and structure of this hybrid zone? (3) Do patterns of hybridization differ between the novel hybrid taxon and *R. ×duclouxii*?

## Results

### Morphometric analysis

**Fig. 3:**
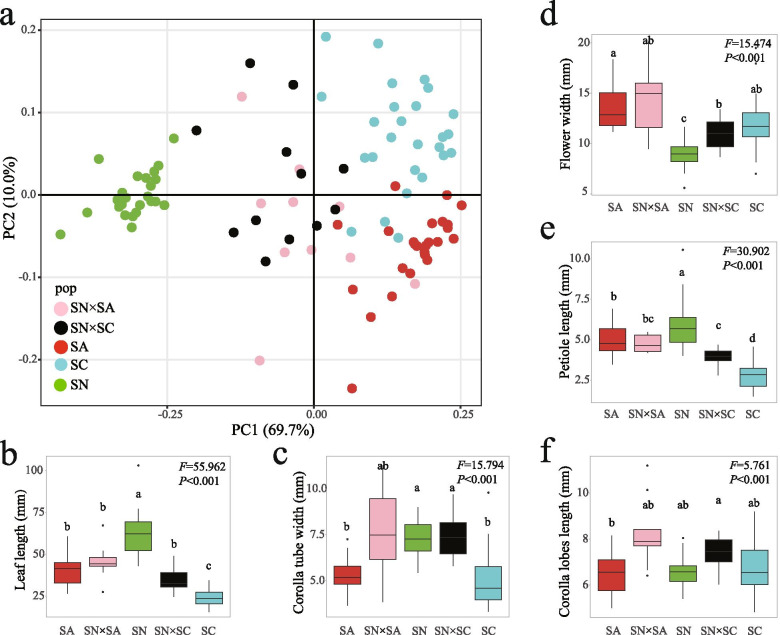
Traits analysis for three parental species of *Rhododendron* and their two types of hybrids. a. PCoA result of total trait variation for 97 samples; b-f. Results of ANOVA analysis for single trait variation of 97 samples (b. leaf length; c. corolla tube width; d. flower width; e. petiole length; f. corolla lobes length). Letters above each of box plots indicate significant differences. (*R. spiciferum* [SC], *R. spinuliferum* [SN], *R. scabrifolium* [SA], *R. ×duclouxii* [SN×SC], the novel hybrid [SN×SA: *R. spinuliferum* × *R. scabrifolium*])

The distribution of variance in the PCoA based on morphological traits of 97 individuals distributed across the five taxa, was 69.7 % 10.0 % and 7.8 % for the first three axes, respectively, resulting in a cumulative value of 87.5 %. Individuals of the three parental species (*R. spiciferum* (SC), *R. spinuliferum* (SN), *R. scabrifolium* (SA)) form distinct clusters (Fig. 3a). For the two hybrid taxa, individuals of *R.* ×*duclouxii* (SN×SC) clustered between the three parent species, while individuals of the putative novel hybrid (SN×SA) were mainly distributed between *R. scabrifolium* and *R. spinuliferum*; hybrid individuals (*R. ×duclouxii* and SN×SA) showed some degree of overlap (Fig. 3a). When we considered variation among parental and hybrid individuals for the 14 quantitative traits separately, some interesting patterns were detected. Firstly, values for individuals of *R. spinuliferum* are larger than both *R. spiciferum* and *R. scabrifolium* for six traits (leaf length, leaf width, leaf area, flower tube length, style length, filaments length) revealing an inverted ‘V’ pattern (*R. spinuliferum* > hybrid > *R. spiciferum*/*R. scabrifolium*) (Fig. 3b, Fig. S1), while other traits (flower tube width, stigma width, ovary width) show a pattern that resembles an inverted ‘U’ (*R. spinuliferum*≈hybrid > *R. spiciferum*/*R. scabrifolium*) (Fig. 3c, Fig. S1). In addition, the flower width of SN*×*SA individuals were similar to that of *R. scabrifolium* individuals, while *R. ×duclouxii* individuals have a flower width that is more similar to individuals of *R. spiciferum* (Fig. 3d). Individuals of *R. scabrifolium* and *R. spinuliferum* had similar petiole length (Fig. 3e), and the two types of hybrid individuals had longer corolla lobes when compared to parental taxa (Fig. 3f). For some flower traits (e.g., style length, stigma width, filaments length), the magnitude of variation among SN*×*SA individuals was greater than those of *R. ×duclouxii* individuals (Fig. S1). The leaf width and pedicel length of SN×SA were significantly larger than *R. ×duclouxii* (Fig S1a, d). For the other quantitative traits, the differences between SN×SA and *R. ×duclouxii* are not significant.

### The genetic structure of the hybrid zone

**Fig. 4:**
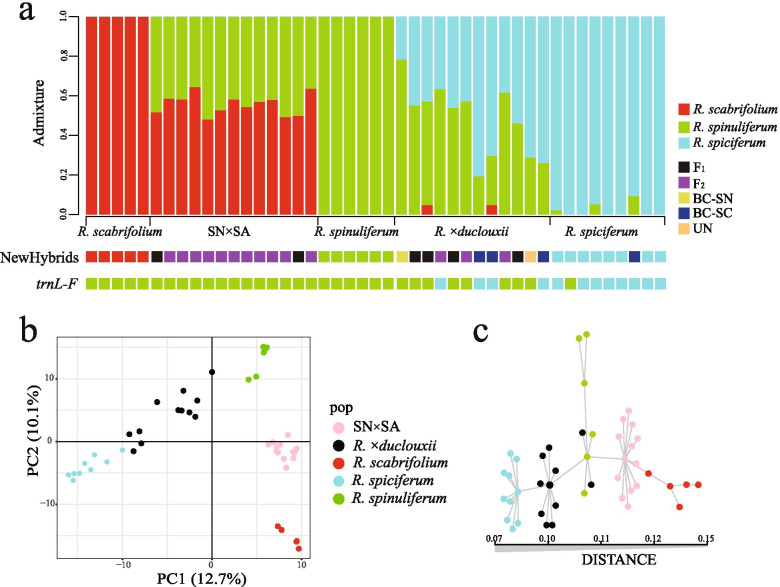
The genetic structure of the three parental species of *Rhododendron* and their two types of hybrids. (a) Results of Admixture analysis (K=3), NewHybrids analysis (BC-SN = backcross with SN, BC-SC = backcross with SC, UN = unidentified) and individual haplotypes for the *trnL-F* cpDNA region; (b) PCoA analysis of the hybrid zone based on the five groups of taxa; (c) MSN analysis showing the genetic distance among individuals within each of the five groups. (*R. spiciferum* [SC], *R. spinuliferum* [SN], *R. scabrifolium* [SA], *R. ×duclouxii* [SN×SC], the novel hybrid [SN×SA: *R. spinuliferum* × *R. scabrifolium*])

Approximately 40 Gb ddRAD-seq raw data was generated for all 45 individuals. After mapping to the *Rhododendron williamsianum* reference genome [50], on average, 617,833 loci (RAD tags) were assembled and the average coverage depth per locus was 27.7×. Furthermore, 30,346 SNPs were retained based on the *populations* pipeline analysis in Stacks. Finally, 11,011 SNPs were identified for Dataset A after Plink filtering. Admixture analysis based on the SNPs in Dataset A, showed that when K=3, the three parental species (*R. spiciferum*, *R. spinuliferum*, *R. scabrifolium*) formed three distinct genetic groups (Fig. 4a), and was the best-fit model. Individuals of SN×SA were a genetic admixture of *R. scabrifolium* and *R. spinuliferum*. Of which five individuals contained approximately 50 % of the genetic component from each parental species, another eight individuals contained a greater genetic proportion of *R. scabrifolium* than *R. spinuliferum* (Fig. 4a). The individuals of *R. ×duclouxii* revealed complex genetic profiles composed of varying amounts of *R. spinuliferum* and *R. spiciferum* genetic admixture (Fig. 4a). No *R. scabrifolium × R. spiciferum* hybrid individuals were detected in the hybrid zone. When K=4 and K=5, neither SN×SA nor *R. ×duclouxii* individuals formed a unique genetic cluster (Fig. S2).

The results of the PCoA analysis based on the SNPs Dataset A were in accordance with the results of the Admixture analysis. The first two principal axes explained 12.7 % and 10.1 % of the variance, respectively (Fig. 4b). The three parental species, and the two types of hybrid taxa (*R. ×duclouxii* and SN×SA), formed five respective well-differentiated clusters (Fig. 4b), which corresponded relatively close to those identified in the PCoA based on morphology. *Rhododendron ×duclouxii* individuals were intermediate between *R. spinuliferum* and *R. spiciferum*, while the individuals of SN×SA were intermediate between *R. spinuliferum* and *R. scabrifolium* (Fig. 4b). There were obvious genetic differences between individuals of SN×SA and *R. ×duclouxii*; the individuals of SN×SA were distinctly separated from the two parents, while individuals of *R. ×duclouxii* were placed on a continuum from *R. spiciferum* to *R. spinuliferum*, although more individuals were closer to *R. spiciferum* (Fig. 4b), which is consistent with the result of the Admixture analysis. Furthermore, no hybrid individuals between *R. scabrifolium* and *R. spiciferum* were detected. Minimum Spanning Network (MSN) analysis showed a similar result to the PCoA, where the three species separated well and the two types of hybrids fell between their parents, although two individuals of *R. ×duclouxii* clustered much closer to *R. spinuliferum* (Fig. 4c).

### Extent and direction of hybridization

NewHybrids results indicated that nearly all the hybrid individuals were divided into a specific class except for one individual of *R. ×duclouxii* (Fig. 4a). For individuals of SN*×*SA, two were assigned to F_1_ class, while others were assigned to F_2_ class, corresponding to the results of the SNPs Admixture analysis (Fig. 4a). The *R. ×duclouxii* individuals were assigned to multiple classes: four were F_1_, three were F_2_, one was backcross to *R. spinuliferum*, three were backcrosses to *R. spiciferum*, and one was unidentified (possible F_3_ or later generation). One individual of *R. spiciferum* was assigned as backcross with *R. spiciferum*, suggestive of a possible hybrid individual, corresponding well to the results of Admixture and PCoA (Fig. 4a).

The sister groups *R. scabrifolium* and *R. spinuliferum* shared the same *trnL-F* haplotype (*R. spinuliferum* haplotype) as shown in Yan et al. [58]. In this study, we found all the individuals of *R. scabrifolium* and SN*×*SA share the *R. spinuliferum trnL-F* haplotype (Fig. 4a). For the *R. ×duclouxii* individuals, four individuals contained the *R. spiciferum* haplotype, while eight comprised the *R. spinuliferum* haplotype. One individual of *R. spiciferum* had the *R. spinuliferum trnL-F* haplotype, but the nDNA genetic structure indicated it was *R. spiciferum* (Fig. 4a). The discrepancy between cpDNA and nDNA for this individual is probably due to hybridization followed by repeated backcrossing with *R. spiciferum*.

## Discussion

### Confirmation of a novel hybrid taxon

In this study, we screened the origin of a putative novel hybrid taxon (SN*×*SA) for 13 individual plants collected from our *Rhododendron* hybrid zone. Morphologically, the majority of these individuals were distributed between *R. scabrifolium* and *R. spinuliferum*, while a few samples were distributed between *R. spiciferum* and *R. spinuliferum* (Figs. 1e and 3a). In addition, the Bayesian genetic clustering results indicated that the genetic composition of SN*×*SA individuals is composed of *R. scabrifolium* and *R. spinuliferum* (Fig. 4a), and the *trnL-F* haplotype analysis showed they share the same maternal chloroplast haplotype as the two parental species [58]. Given that all three parental species (*R. spiciferum*, *R. spinuliferum R. scabrifolium*) formed independent clusters based on either morphology or ddRAD data, collectively our results indicate a novel hybrid taxon (SN*×*SA) is present at our study site and is of *R. scabrifolium* × *R. spinuliferum* origin. This result represents a first step to understand the potential evolutionary effects of natural hybridization among *Rhododendron* taxa within our hybrid zone, where further study on the ecological and genetic mechanisms (e.g., fitness differences, dispersal ability, reproductive barriers) governing formation and persistence of these novel hybrid taxa is needed [21, 33, 59, 60].

### Complex hybridization among three closely related species

Our morphological and genetic results indicate that the hybrid zone is a complex hybrid swarm from two types of hybrid taxa among three closely related species. Our study site is located in one of the species diversification and distribution centers of *Rhododendron* in China, where most of the species were formed in Pliocene and Quaternary (< 5 Ma) due to adaptive radiation and subsequent rapid evolution [49, 53, 61–63]. In addition, this region contains many natural hybrid zones of *Rhododendron* species [13, 58, 64–68]. At our study site, which intersects the distribution range of our three parental species [16, 58], it would appear that gene flow among *R. scabrifolium*, *R. spiciferum* and *R. spinuliferum* is possible, although we found no evidence for the formation of hybrids between the former two species. Our previous studies show that post-zygotic reproductive barriers between *R. spinuliferum* and *R. spiciferum* are weak [69], and given that *R. scabrifolium* is the sister taxon of *R. spinuliferum* [58], it seems reasonable that the three species can hybridize with each other, although only two types of hybrid taxa were detected at the study site.

Based on field observations, the lack of hybrids produced between *R. scabrifolium* and *R. spiciferum* may be due to strong pre-zygotic barriers to reproduction. At our study site, *R. scabrifolium* and *R. spiciferum* appear to occupy different micro-environments: *R. scabrifolium* grows in lush evergreen broad-leaf forest, whereas *R. spiciferum* prefers to grow in more open disturbed habitats (Fig. 2; Table S1). In addition, the two species were often found in different plots within the hybrid zone, which may also reduce pollination opportunities, and subsequent gene flow, between *R. scabrifolium* and *R. spiciferum* at our study site. Thus, ecological niche divergence may play an important role for our study species, and this has also been found in other species (e.g., *Silene* [70], *Senecio* [71]). Based on our observations, the flowering phenology is also different between *R. scabrifolium* and *R. spiciferum* in the hybrid zone; peak anthesis for *R. scabrifolium* is in mid-February, whereas *R. spiciferum* typically flowers mid-March. Taken together, these factors may form a strong reproductive barrier between *R. scabrifolium* and *R. spiciferum*. Although we acknowledge that another possible reason for the lack of hybrids produced between *R. scabrifolium* and *R. spiciferum* may due to sampling bias (i.e., more sampling may increase the chances of finding such hybrids), these plausible barriers to gene flow could also explain why we didn’t find hybrid individuals between *R. scabrifolium* and *R. spiciferum* at our study site. Pollination experiments and further studies are needed to assess the magnitude of pre- and post-zygotic barriers to hybrid formation among the parental taxa.

### Hybrid zone structure

Results of the PCoA and ANOVA analysis of morphological traits indicated that *R. ×duclouxii* and SN×SA share some similar morphological characters (Figs. 1 and 3, S1), which may shed light on the genetic architecture of shared phenotypic traits among the hybrid taxa present in the hybrid zone [72]. However, our genetic data revealed that the genetic structure of these two types of hybrids is quite different. *Rhododendron ×duclouxii* individuals consisted of multiple kinds of offspring, which include not only F_1_ individuals, but also backcrosses and F_2_ individuals, whereas SN×SA individuals were only composed of F_1_ and F_2_ offspring, and no backcross individuals were detected. In addition, we found more *R.* ×*duclouxii* individuals have the *R. spinuliferum trnL-F* sequence compared to those with *R. spiciferum*. This result indicates that for the formation of *R. ×duclouxii* hybrids is bidirectional but asymmetric between *R. spinuliferum* and *R. spiciferum*, which was also reported in our previous studies [58, 69]. For the formation of the novel hybrid taxon (SN×SA), we could not ascertain the symmetry or direction of hybridization because the sequence for the trn*L-F* region, and even for the whole plastid genome (unpublished data), of *R. scabrifolium* is same as that for *R. spinuliferum* [58]. Taken together, it seems likely that our hybrid zone represents a mosaic model, where different classes and generations of hybrids are selected by different microhabitats in areas of sympatry among the parental species at our study site, although we acknowledge that more environmental data, and fitness assessments, are necessary to verify this hypothesis. While this model is common in herbaceous perennial plants, e.g. *Aquilegia* [29, 73], *Senecio* [30], *Petunia* [74], its occurrence in hybridizing woody plant taxa is rare and requires additional evidence.

Although determining the structure of hybrid zones is essential for discerning the mechanisms and potential evolutionary outcomes of hybridization [33, 75, 76], systems that involve three or more parental taxa can make this process particularly challenging, especially when comparing results among studies. For example, three species of *Ligularia* (*L. duciformis*, *L. yunnanensis* and *L. cyathiceps*) are known to hybridize and form two groups of hybrids, a result that is similar to our findings, but unlike our study, both groups of the *Ligularia* hybrid taxa are restricted to F_1_ with no history of gene introgression [18]. Although in our study the two groups of hybrid taxa shared *R. spinuliferum* as one of their parental species, they display a different pattern in population structure than that found in *Ligularia* [18]. When compared to our study, the degree of reproductive isolation between parental taxa has been shown to be weak in both this study and our previous studies [58, 69], it seems likely that, *R.* ×*duclouxii* is in close proximity, may contribute to the formation of complex hybrid swarm in the future. Other hybrids zones that include three hybridizing parental species have shown that recurrent gene flow can induce adaptive introgression [43, 77–79]. Our study system represents a novel opportunity to explore mechanisms of potential adaptive introgression in *Rhododendron*, where further empirical study may determine whether the presence of novel hybrid taxa at our study site is the result of reproductive or ecological isolation resulting in unique genetic architectures that influence the adaption of hybrids in novel habitats. Further study, with increased sampling within our hybrid zone, may enable us to uncover the ecological and evolutionary mechanisms that ultimately contribute to hybrid speciation in the genus *Rhododendron*.

## Conclusions

Our results uncover the presence of a complex hybrid zone in Yunnan, SW China that is comprised of three parental *Rhododendron* species (*R. scabrifolium, R. spinuliferum*, *R. spiciferum)* and two types of hybrid taxa, including a novel hybrid taxon of *R. scabrifolium* × *R. spinuliferum* recognized for the first time. Although the two types of hybrid taxa found at our site share *R. spinuliferum* as one of the parents, hybrid genetic admixture varies among them; individuals of *R.* ×*duclouxii* are an admixture of F_1_, F_2_, and backcrosses, while the novel hybrids are dominated by F_2_, with a low proportion of F_1_ and no backcross individuals. This study represents a unique opportunity to dissect ecological and evolutionary mechanisms associated with adaptive introgression, where hybridization among three parental taxa can result in the formation of complex hybrid swarms that consist of hybrid taxa that vary in their evolutionary potentials.

## Methods

### Study site and plant sampling

The hybrid zone is located in Nanhua County, Chuxiong Prefecture, Yunnan Province, southwest China (Fig. 2). Three closely related species in the *Rhododendron* subsect. *Scabrifolia* (*R. scabrifolium*, *R. spiciferum*, and *R. spinuliferum*) and their hybrids were found in sympatry within this hybrid zone. Voucher specimens (two to three duplicates per individual) and DNA samples (healthy leaves dried immediately with silica-gel) were collected from the wild. A total of 45 individuals were sampled: nine individuals of *R. spiciferum*, six of *R. spinuliferum*, five of *R. scabrifolium*, 12 individuals of *R. ×duclouxii*, and 13 individuals of the putative novel hybrid taxon (SN×SA) (Table S1). Total genomic DNA was extracted using the modified CTAB method [80]. All specimens were identified based on morphology, and were deposited in the herbarium of Kunming Institute of Botany (KUN), Chinese Academy of Sciences, Kunming, China.

### ddRAD-seq and cpDNA sequencing

Library construction of double digest restriction-site-associated DNA sequencing (ddRAD-seq) was according to the protocol of Yang et al. [81]. Genomic DNA of each individual was digested with two restriction enzymes (*AvaII*, NEB Cat#: R0153S; *MspI*, NEB Cat#: R0106S), and then size-selected to a range of 500-700 bp. Library sequencing was performed on an Illumina HiSeq X Ten (San Diego, CA, USA) with 150 bp paired-end reads by Cloud Health (Shanghai, China). Libraries were pooled to a target of approximately 1.2 Gb raw data per individual. To determine cpDNA haplotypes, the chloroplast DNA region *trnL-F* was sequenced for all 45 sampled individuals from the hybrid zone. The protocols of PCR and sequencing followed Yan et al. [58].

### Morphometric data collection

To examine morphological differentiation among *R. spiciferum*, *R. spinuliferum*, *R. scabrifolium*, and the two types of hybrid taxa (*R. ×duclouxii* and SN×SA), we chose 41 of the 45 individuals to measure 23 morphological traits (Table S2), which include nine traits that are qualitative (calyx lobe conspicuous/inconspicuous; density of abaxial and adaxial leaf hairs; density of corolla scales; filament hair present/absent; style hair present/absent; flower color; stigma color; anther color) and 14 quantitative traits (leaf length, width, thickness, and area; petiole length; pedicel length; corolla lobe length; corolla tube width and length; flower width; style length; filament length; stigma width; ovary width). For each individual, a total of three leaves and three flowers, without obvious symptoms of pathogen or physical damage, were selected for trait measurement. Qualitative floral traits were assessed by visual inspection at the site, while quantitative floral traits were measured by vernier caliper; leaf area measurements were based on scanned images (CanonScan LiDE 220; Canon, Tokyo, Japan). In addition, the 23 morphological traits were measured for 19 individuals of *R. spinuliferum*, 20 individuals of *R. scabrifolium*, and 17 individuals of *R. spiciferum* from other allopatric populations (close to the hybrid zone) to supplement the dataset.

### Data analysis

To assess the dispersion of morphological traits in multivariate trait space, Principal Coordinates Analysis (PCoA) for morphometric distance (i.e., Gower distance) was conducted in the R package *ggfortify* [82]. Variations of each quantitative trait were shown in boxplots generated by the R program [83]. ANOVA analysis was performed for each quantitative trait using SPSS v19 [84].

The ddRAD sequencing data was analyzed by Stacks v.2.04 [85]. In the first step, raw reads were de-multiplexed and quality-filtered by using the pipeline *process_radtags*. FastQC v.0.11.4 [86] was used to assess read quality and GC-content. All reads were then trimmed to 140 bp to remove low quality bases by using -t parameter in Stacks v.2.04. The genome of *R. williamsianum* [50] was used as a reference in the reference-based analyses pipeline. To obtain a large number of high-quality SNPs, we treated the three *Rhododendron* species, and the two hybrid taxa (*R. ×duclouxii* and SN×SA), as five different populations in the *populations* pipeline. Loci were filtered in two steps. Firstly, the number of SNPs were reduced based on the following parameters: write_single_snp (restrict data analysis to only the first SNP per locus), r = 0.7 (a locus must exist in at least 70 % of the individuals for each of the populations), p = 5 (a locus should be present in each of the five populations), and min-maf = 0.05 (minimum minor allele frequency required to process a nucleotide site at a locus) [18]. After this reduction step, SNPs with data missing call rates exceeding 0.2 (geno=0.2), and a Hardy-Weinberg equilibrium exact test p-value below 0.01 (hwe=0.01), were further filtered with Plink v1.90 [87]. All SNPs generated from these two filtering steps were used as Dataset A. To distinguish hybrids in the NewHybrids analysis, another dataset (Dataset_B) was screened by the top 400 highest species pairwise *F*_ST_ unlinked SNPs using the function getTopLoc() in the *hybriddetective* R package [88, 89].

To uncover the genetic structure of the hybrid zone, Dataset A was analyzed by a Bayesian genetic clustering approach using Admixture v.1.3.0 [90] and the best K value (number of genetic clusters ranging from 2 to 7) was evaluated by the CV (cross-validation) value in the log file. To visualize the genetic similarities among the five taxa, Principal Coordinate Analysis (PCoA) was performed using the *ape* package in R [91]. The MSN (Minimum Spanning Network) analysis for all individuals was conducted by *poppr* package in R [92] and the graphs were produced using the *ggplot2* package v3.3.3 [93].

We used NewHybrids v.1.1 to calculate the probability of each individual belonging to a particular genotypic class (P1, P2, F1, F2, BC1, BC2) [94]. Since we identified a priori two different types of hybrid taxa in this hybrid zone, NewHybrids analysis was based on two different SNPs Datasets (high *F*_ST_): Dataset_B1 includes all individuals from *R. spinuliferum*, *R. scabrifolium* and SN×SA, and Dataset_B2 includes all individuals from *R. spinuliferum*, *R. spiciferum* and *R. ×duclouxii*. This analysis was run for 100,000 rounds after a 100,000 burn-in iteration. If an individual was assigned to a genotype class with a probability ≥ 0.9, this individual was regarded as belonging to that class.

To detect the *trnL-F* cpDNA haplotype, *trnL-F* sequences of all individuals within the hybrid zone were aligned in Geneious v.8.1 [95]. The informative sites were summarized, and the haplotypes belonging to *R. spiciferum* and *R. spinuliferum* were then assigned following Yan et al. [58].

## Supplementary Information

**Additional file 1.**

## Data Availability

All newly generated *trnL-F* sequences had been deposited in the NCBI with the GenBank accession numbers MZ493237 to MZ493279 (Table S1). Raw ddRAD-seq reads are available at the National Center for Biotechnology Information (NCBI) database (https://www.ncbi.nlm.nih.gov) under BioProject ID PRJNA746356 (can be viewed at https://dataview.ncbi.nlm.nih.gov/object/PRJNA746356?reviewer=vls9t8u2codbav11mo4pnugsna and will be released after publication). The morphological data are provided in Table S3.

## Abbreviations

ddRAD: double digest Restriction-site Associated DNA
SN: *R. spinuliferum*
SA: *R. scabrifolium*
SC: *R. spiciferum*
SW: Southwest
SN×SC: *R. spinuliferum* × *R. spiciferum*
SN×SA: *R. spinuliferum* × *R. scabrifolium*
PCoA: Principal Coordinate Analysis
SNP: Single Nucleotide Polymorphism
MSN: Minimum Spanning Network
