# Supplementary material for: Natural hybridization among three Rhododendron species (Ericaceae) revealed by morphological and genomic evidence
Source: BMC Plant Biol. 2021 Nov 11;21:529. doi: 10.1186/s12870-021-03312-y (PMC8582147; doi:10.1186/s12870-021-03312-y)
Supplement: Supplementary file 1 — Additional file 1. [file 12870_2021_3312_MOESM1_ESM.zip › Supporting Document_Table S1S2 and Figure S1S2_ESM.docx]

**Natural hybridization among three *Rhododendron* species (Ericaceae) revealed by morphological and genomic evidence**

Wei Zheng^1,2#^, Li-Jun Yan^1,2,3#^, Kevin S. Burgess^4^, Ya-Huang Luo^1^, Jia-Yun Zou^1,2^, Han-Tao Qin^1,2^, Ji-Hua Wang^5*^, Lian-Ming Gao^1,6*^

^1^*CAS Key Laboratory for Plant Diversity and Biogeography of East Asia,* *Kunming Institute of Botany, Chinese Academy of Sciences, Kunming, Yunnan 650201, China*

^2^*University of Chinese Academy of Sciences, Beijing 10049, China*

^3^*College of Vocational and Technical Education, Yunnan Normal University, Kunming, Yunnan 650092, China*

^4^*Department of Biology, Columbus State University, University System of Georgia, Columbus, GA, USA 31907-5645*

*^5^The Flower Research Institute, Yunnan Academy of Agricultural Sciences, Kunming 650205, China*

^6^*Lijiang Forest Biodiversity National Observation and Research Station, Kunming Institute of Botany, Chinese Academy of Sciences, Lijiang 674100, Yunnan, China*

*Corresponding authors: LM Gao: gaolm@mail.kib.ac.cn; JH Wang: [wjh0505@gmail.com](mailto:wjh0505@gmail.com)

^#^WZ & LJY contributed equally to this manuscript.

Table S1. Sampling information for all 97 individuals of these five taxa of *Rhododendron*. All samples (*Rhododendron* *scabrifolium*, *R. spinuliferum*, *R. spiciferum*, *R. ×duclouxii*, novel hybrid taxa (SN×SA)) were collected from Yunnan province, southwest China.

| Voucher Number | Taxon | Sampling location | Altitude (m) | Plot | Habitat | GenBank accession number of *trnL-F* sequence |
| --- | --- | --- | --- | --- | --- | --- |
| ZW-170375 | *R. scabrifolium* | N 25°17′24.4″, E 101°05′01.7″ | 2049 | Plot 2 | dense forest of Fagaceae | MZ493235 |
| ZW-170384 | *R. scabrifolium* | N 25°17′23.9″ E 101°05′00.4″ | 2093 | Plot 2 | dense forest of Fagaceae | MZ493236 |
| ZW-171093 | *R. scabrifolium* | N 25°17′24.5″ E 101°05′01.0″ | 2049 | Plot 2 | dense forest of Fagaceae | MZ493237 |
| ZW-171096 | *R. scabrifolium* | N 25°17′25.4″ E 101°05′01.6″ | 2036 | Plot 2 | dense forest of Fagaceae | MZ493238 |
| ZW-171097 | *R. scabrifolium* | N 25°17′25.8″ E 101°05′01.3″ | 2038 | Plot 2 | dense forest of Fagaceae | MZ493239 |
| ZW-170378 | SN*×*SA | N 25°17′24.4″ E 101°05′01.3″ | 2067 | Plot 2 | dense forest of Fagaceae | MZ493254 |
| ZW-170390 | SN*×*SA | N 25°14′03.9″ E 101°05′34.7″ | 2077 | Plot 4 | sparse pine forest | MZ493275 |
| ZW-170391 | SN*×*SA | N 25°14′03.4″ E 101°05′34.7″ | 2079 | Plot 4 | sparse pine forest | MZ493276 |
| ZW-170392 | SN*×*SA | N 25°14′03.1″ E 101°05′35.2″ | 2090 | Plot 4 | sparse pine forest | MZ493277 |
| ZW-170395 | SN*×*SA | N 25°14′02.4″ E 101°05′36.6″ | 2095 | Plot 4 | sparse pine forest | MZ493278 |
| ZW-171099 | SN*×*SA | N 25°14′03.5″ E 101°05′33.6″ | 2056 | Plot 4 | sparse pine forest | MZ493279 |
| YLJ-180083 | SN*×*SA | N 25°14′03.2″E 101°05′35.3″ | 2200 | Plot 4 | sparse pine forest | MZ493269 |
| YLJ-180078 | SN*×*SA | N 25°14′03.2″E 101°05′35.3″ | 2200 | Plot 4 | sparse pine forest | MZ493268 |
| YLJ-180075 | SN*×*SA | N 25°14′03.2″E 101°05′35.3″ | 2200 | Plot 4 | sparse pine forest | MZ493267 |
| YLJ-12680 | SN*×*SA | N 25°15'47.5" E 101°06'25.6" | 1974 | Plot 3 | sparse coniferous mixed forest | MZ493272 |
| YLJ-12672 | SN*×*SA | N 25°15'47.4" E 101°06'25.7" | 1956 | Plot 3 | sparse coniferous mixed forest | MZ493271 |
| YLJ-180097 | SN*×*SA | N 25°18′12.7″ E 101°4′6.3″ | 2203 | Plot 1 | sparse pine forest | MZ493270 |
| YLJ-12687 | SN*×*SA | N 25°15'47.3" E 101°06'25.5" | 1962 | Plot 3 | sparse coniferous mixed forest | MZ493273 |
| YLJ-12686 | *R. spinuliferum* | N 25°15'47.5" E 101°06'25.5" | 1951 | Plot 3 | sparse coniferous mixed forest | MZ493249 |
| ZW-170399 | *R. spinuliferum* | N 25°14′02.9″ E 101°05′36.7″ | 2083 | Plot 4 | sparse pine forest | MZ493253 |
| ZW-170376 | *R. spinuliferum* | N 25°17′24.4″ E 101°05′01.5″ | 2055 | Plot 4 | sparse pine forest | MZ493250 |
| ZW-170380 | *R. spinuliferum* | N 25°17′24.1″ E 101°05′00.4″ | 2073 | Plot 2 | dense forest of Fagaceae | MZ493251 |
| ZW-170382 | *R. spinuliferum* | N 25°17′23.5″ E 101°04′59.6″ | 2082 | Plot 2 | dense forest of Fagaceae | MZ493252 |
| ZW-171098 | *R. spinuliferum* | N 25°17′24.10″ E 101°05′01.9″ | 2073 | Plot 2 | dense forest of Fagaceae | MZ493254 |
| ZW-170402 | *R. ×duclouxii* | N 25°12′37.9″ E 101°09′14.4″ | 2021 | Plot 5 | open shrub with pine trees | MZ493257 |
| ZW-170403 | *R. ×duclouxii* | N 25°12′38.2″ E 101°09′13.9″ | 2020 | Plot 5 | open shrub with few pine trees | MZ493258 |
| ZW-170409 | *R. ×duclouxii* | N 25°13′52.9″ E 101°11′11.5″ | 1932 | Plot 6 | slope surrounding by farmland | MZ493262 |
| ZW-170401 | *R. ×duclouxii* | N 25°12′37.7″ E 101°09′14.6″ | 2024 | Plot 5 | open shrub with few pine trees | MZ493256 |
| ZW-170405 | *R. ×duclouxii* | N 25°12′38.3″ E 101°09′12.8″ | 2017 | Plot 5 | open shrub with few pine trees | MZ493259 |
| ZW-170406 | *R. ×duclouxii* | N 25°12′37.9″ E 101°09′09.3″ | 2010 | Plot 5 | open shrub with few pine trees | MZ493260 |
| ZW-170408 | *R. ×duclouxii* | N 25°13′52.4″ E 101°11′13.7″ | 1936 | Plot 6 | slope surrounding by farmland | MZ493261 |
| ZW-170411 | *R. ×duclouxii* | N 25°13′51.7″ E 101°11′09.5″ | 1933 | Plot 6 | slope surrounding by farmland | MZ493263 |
| ZW-170400 | *R. ×duclouxii* | N 25°12′38.2″ E 101°09′14.9″ | 2026 | Plot 5 | open shrub with few pine trees | MZ493255 |
| ZW-171100 | *R. ×duclouxii* | N 25°12′38.3″ E 101°09′14.8″ | 1999 | Plot 5 | open shrub with few pine trees | MZ493264 |
| ZW-171103 | *R. ×duclouxii* | N 25°12′38.0″ E 101°09′14.3″ | 2001 | Plot 5 | open shrub with few pine trees | MZ493266 |
| ZW-171101 | *R. ×duclouxii* | N 25°12′38.1″ E 101°09′15.0″ | 2000 | Plot 5 | open shrub with few pine trees | MZ493265 |
| ZW-171106 | *R. spiciferum* | N 25°12′38.1″ E 101°09′14.4″ | 1998 | Plot 5 | open shrub with few pine trees | MZ493241 |
| ZW-171110 | *R. spiciferum* | N 25°12′38.7″ E 101°09′13.5″ | 1995 | Plot 5 | open shrub with few pine trees | MZ493242 |
| ZW-171119 | *R. spiciferum* | N 25°12′33.7″ E 101°09′02.3″ | 1967 | Plot 5 | open shrub with few pine trees | MZ493247 |
| ZW-170407 | *R. spiciferum* | N 25°13′52.9″ E 101°11′14.7″ | 1935 | Plot 5 | open shrub with few pine trees | MZ493240 |
| ZW-171112 | *R. spiciferum* | N 25°12′38.9″ E 101°09′13.0″ | 1991 | Plot 5 | open shrub with few pine trees | MZ493243 |
| ZW-171114 | *R. spiciferum* | N 25°12′34.3″ E 101°09′02.4″ | 1963 | Plot 5 | open shrub with few pine trees | MZ493244 |
| ZW-171116 | *R. spiciferum* | N 25°12′34.3″ E 101°09′02.3″ | 1967 | Plot 5 | open shrub with few pine trees | MZ493245 |
| ZW-171117 | *R. spiciferum* | N 25°12′34.1″ E 101°09′02.1″ | 1968 | Plot 5 | open shrub with few pine trees | MZ493246 |
| ZW-171124 | *R. spiciferum* | N 25°12′33.6″ E 101°09′01.6″ | 1964 | Plot 5 | open shrub with few pine trees | MZ493248 |
| ZW190450 | *R. scabrifolium* | N 26°1′20.99″ E 101°5′19.97″ | 2154 | DaYao country | destructed shrub |  |
| ZW190452 | *R. scabrifolium* | N 26°1′20.99″ E 101°5′19.97″ | 2154 | DaYao country | destructed shrub |  |
| ZW190454 | *R. scabrifolium* | N 26°1′20.99″ E 101°5′19.97″ | 2154 | DaYao country | destructed shrub |  |
| ZW190455 | *R. scabrifolium* | N 26°1′20.99″ E 101°5′19.97″ | 2154 | DaYao country | destructed shrub |  |
| ZW190457 | *R. scabrifolium* | N 26°1′20.99″ E 101°5′19.97″ | 2154 | DaYao country | destructed shrub |  |
| ZW190458 | *R. scabrifolium* | N 26°1′20.99″ E 101°5′19.97″ | 2154 | DaYao country | destructed shrub |  |
| ZW190461 | *R. scabrifolium* | N 26°1′20.99″ E 101°5′19.97″ | 2154 | DaYao country | destructed shrub |  |
| ZW190464 | *R. scabrifolium* | N 26°1′20.99″ E 101°5′19.97″ | 2154 | DaYao country | destructed shrub |  |
| ZW190465 | *R. scabrifolium* | N 26°1′20.99″ E 101°5′19.97″ | 2154 | DaYao country | destructed shrub |  |
| ZW190467 | *R. scabrifolium* | N 26°1′20.99″ E 101°5′19.97″ | 2154 | DaYao country | destructed shrub |  |
| ZW190471 | *R. scabrifolium* | N 25°21′47.42″ E 101°5′19.97″ | 2150 | YaoAn country | dense forest of Fagaceae |  |
| ZW190473 | *R. scabrifolium* | N 25°21′47.42″ E 101°5′19.97″ | 2150 | YaoAn country | dense forest of Fagaceae |  |
| ZW190474 | *R. scabrifolium* | N 25°21′47.42″ E 101°5′19.97″ | 2150 | YaoAn country | dense forest of Fagaceae |  |
| ZW190475 | *R. scabrifolium* | N 25°21′47.42″ E 101°5′19.97″ | 2150 | YaoAn country | dense forest of Fagaceae |  |
| ZW190477 | *R. scabrifolium* | N 25°21′47.42″ E 101°5′19.97″ | 2150 | YaoAn country | dense forest of Fagaceae |  |
| ZW190478 | *R. scabrifolium* | N 25°21′47.42″ E 101°5′19.97″ | 2150 | YaoAn country | dense forest of Fagaceae |  |
| ZW190479 | *R. scabrifolium* | N 25°21′47.42″ E 101°5′19.97″ | 2150 | YaoAn country | dense forest of Fagaceae |  |
| ZW190487 | *R. scabrifolium* | N 25°21′47.42″ E 101°5′19.97″ | 2150 | YaoAn country | dense forest of Fagaceae |  |
| ZW190488 | *R. scabrifolium* | N 25°21′47.42″ E 101°5′19.97″ | 2150 | YaoAn country | dense forest of Fagaceae |  |
| ZW190489 | *R. scabrifolium* | N 25°21′47.42″ E 101°5′19.97″ | 2150 | YaoAn country | dense forest of Fagaceae |  |
| ZW-170413 | *R. spinuliferum* | N 24°40′48.9″ E 101°39′22.4″ | 1924 | ShuangBai country | dense forest of Fagaceae |  |
| ZW-170414 | *R. spinuliferum* | N 24°40′48.9″ E 101°39′22.4″ | 1924 | ShuangBai country | dense forest of Fagaceae |  |
| ZW-170418 | *R. spinuliferum* | N 24°40′49.8″ E 101°39′14.4″ | 2004 | ShuangBai country | dense forest of Fagaceae |  |
| ZW-170420 | *R. spinuliferum* | N 24°40′49.0″ E 101°39′14.4″ | 2022 | ShuangBai country | dense forest of Fagaceae |  |
| ZW-170424 | *R. spinuliferum* | N 24°40′48.2″ E 101°39′12.4″ | 2025 | ShuangBai country | dense forest of Fagaceae |  |
| ZW-170425 | *R. spinuliferum* | N 24°40′30.1″ E 101°39′46.7″ | 1916 | ShuangBai country | dense forest of Fagaceae |  |
| ZW-170426 | *R. spinuliferum* | N 24°40′30.9″ E 101°39′46.5″ | 1921 | ShuangBai country | dense forest of Fagaceae |  |
| ZW-170427 | *R. spinuliferum* | N 24°40′30.3″ E 101°39′46.0″ | 1920 | ShuangBai country | dense forest of Fagaceae |  |
| ZW-170430 | *R. spinuliferum* | N 24°40′32.5″ E 101°39′46.0″ | 1907 | ShuangBai country | dense forest of Fagaceae |  |
| ZW-170432 | *R. spinuliferum* | N 24°40′32.8″ E 101°39′45.6″ | 1906 | ShuangBai country | dense forest of Fagaceae |  |
| ZW-170565 | *R. spinuliferum* | N 25°28′27.2″ E 103°30′43.7″ | 1906 | MaLong country | dense forest of Fagaceae |  |
| ZW-170568 | *R. spinuliferum* | N 25°28′27.2″ E 103°30′42.8″ | 1993 | MaLong country | dense forest of Fagaceae |  |
| ZW-170570 | *R. spinuliferum* | N 25°28′27.2″ E 103°30′41.1″ | 1997 | MaLong country | dense forest of Fagaceae |  |
| ZW-170573 | *R. spinuliferum* | N 25°28′27.5″ E 103°30′38.7″ | 1992 | MaLong country | dense forest of Fagaceae |  |
| ZW-170574 | *R. spinuliferum* | N 25°28′27.3″ E 103°30′40.4″ | 1999 | MaLong country | dense forest of Fagaceae |  |
| ZW-170577 | *R. spinuliferum* | N 25°28′25.7″ E 103°30′47.9″ | 1994 | MaLong country | dense forest of Fagaceae |  |
| ZW-170578 | *R. spinuliferum* | N 25°28′25.6″ E 103°30′47.1″ | 1994 | MaLong country | dense forest of Fagaceae |  |
| ZW-170580 | *R. spinuliferum* | N 25°28′25.1″ E 103°30′50.3″ | 2007 | MaLong country | dense forest of Fagaceae |  |
| ZW-170581 | *R. spinuliferum* | N 25°28′25.4″ E 103°30′48.5″ | 1997 | MaLong country | dense forest of Fagaceae |  |
| ZW-170582 | *R. spiciferum* | N 24°27′36.5″ E 102°50′19.4″ | 1792 | JiangChuan country | border of open forest |  |
| ZW-170583 | *R. spiciferum* | N 24°27′36.6″ E 102°50′19.1″ | 1783 | JiangChuan country | border of open forest |  |
| ZW-170586 | *R. spiciferum* | N 24°27′36.7″ E 102°50′17.2″ | 1784 | JiangChuan country | border of open forest |  |
| ZW-170587 | *R. spiciferum* | N 24°27′37.4″ E 102°50′20.7″ | 1775 | JiangChuan country | border of open forest |  |
| ZW-170588 | *R. spiciferum* | N 24°27′37.5″ E 102°50′21.1″ | 1772 | JiangChuan country | border of open forest |  |
| ZW-170591 | *R. spiciferum* | N 24°27′37.7″ E 102°50′21.7″ | 1769 | JiangChuan country | border of open forest |  |
| ZW-170592 | *R. spiciferum* | N 24°27′37.3″ E 102°50′22.2″ | 1772 | JiangChuan country | border of open forest |  |
| ZW-170596 | *R. spiciferum* | N 24°27′52.2″ E 102°50′44.6″ | 1861 | JiangChuan country | border of open forest |  |
| ZW-170598 | *R. spiciferum* | N 24°27′48.6″ E 102°50′44.2″ | 1874 | JiangChuan country | border of open forest |  |
| ZW-170600 | *R. spiciferum* | N 24°27′48.5″ E 102°50′44.6″ | 1880 | JiangChuan country | border of open forest |  |
| ZW-170605 | *R. spiciferum* | N 24°33′02.9″ E 102°34′26.7″ | 1980 | JinNing country | open shrub |  |
| ZW-170606 | *R. spiciferum* | N 24°33′03.2″ E 102°34′26.6″ | 1995 | JinNing country | open shrub |  |
| ZW-170607 | *R. spiciferum* | N 24°33′02.7″ E 102°34′26.2″ | 2005 | JinNing country | open shrub |  |
| ZW-170612 | *R. spiciferum* | N 24°33′01.9″ E 102°34′26.2″ | 2025 | JinNing country | open shrub |  |
| ZW-170614 | *R. spiciferum* | N 24°33′02.8″ E 102°34′25.3″ | 2039 | JinNing country | open shrub |  |
| ZW-170618 | *R. spiciferum* | N 24°33′02.8″ E 102°34′23.9″ | 2046 | JinNing country | open shrub |  |
| ZW-170622 | *R. spiciferum* | N 24°33′02.3″ E 102°34′22.9″ | 2053 | JinNing country | open shrub |  |

Table S2. The information of traits used to assess morphological variations among parental and hybrid *Rhododendron* taxa.

| Traits | | Description |  |  |  |  |
| --- | --- | --- | --- | --- | --- | --- |
| qualitative | Calyx lobe | Inconspicuous  (0) | Conspicuous  (1) |  |  |  |
|  | Adaxial leaf hairs | Absent  (0) | Nearly glabrous  (1) | Sparse  (2) | Medium dense  (3) | Dense  (4) |
|  | Abaxial leaf hairs | Absent  (0) | Nearly glabrous  (1) | Sparse  (2) | Medium dense  (3) | Dense  (4) |
|  | Corolla scales | Absent  (0) | Scaly  (1) | Sparse  (2) | Medium dense  (3) | Dense  (4) |
|  | Filament hairs | Absent  (0) | Present  (1) |  |  |  |
|  | Style hairs | Absent  (0) | Present  (1) |  |  |  |
|  | Flower color | White  (1) | Pink-white  (2) | Pink  (3) | Red  (4) |  |
|  | Stigma color | Olivine  (1) | Light yellow  (2) | Yellow  (3) | Red  (4) |  |
|  | Anther color | Purplish black  (1) | Brown  (2) | Purplish red  (3) |  |  |
| quantitative | Leaf length | Length of the leaf blade | | | | |
|  | Leaf width | Width of the leaf blade at the widest point | | | | |
|  | Leaf thickness | Average thickness of the leaf blade | | | | |
|  | Petiole length | Length of the petiole | | | | |
|  | Leaf area | Area of the leaf (include petiole) | | | | |
|  | Pedicel length | Length of the pedicel | | | | |
|  | Corolla lobes length | Length of corolla lobes at the longest point | | | | |
|  | Corolla tube length | Length of corolla tube at the longest point | | | | |
|  | Flower width | Width of the flower at the widest point | | | | |
|  | Corolla tube width | Width of corolla tube at the widest point | | | | |
|  | Style length | Length of style (top of ovary to top of stigma) | | | | |
|  | Filaments length | Length of filaments (bottom of filament to bottom of anther) | | | | |
|  | Stigma width | Stigma width at the widest point | | | | |
|  | Ovary width | Ovary width at the widest point | | | | |


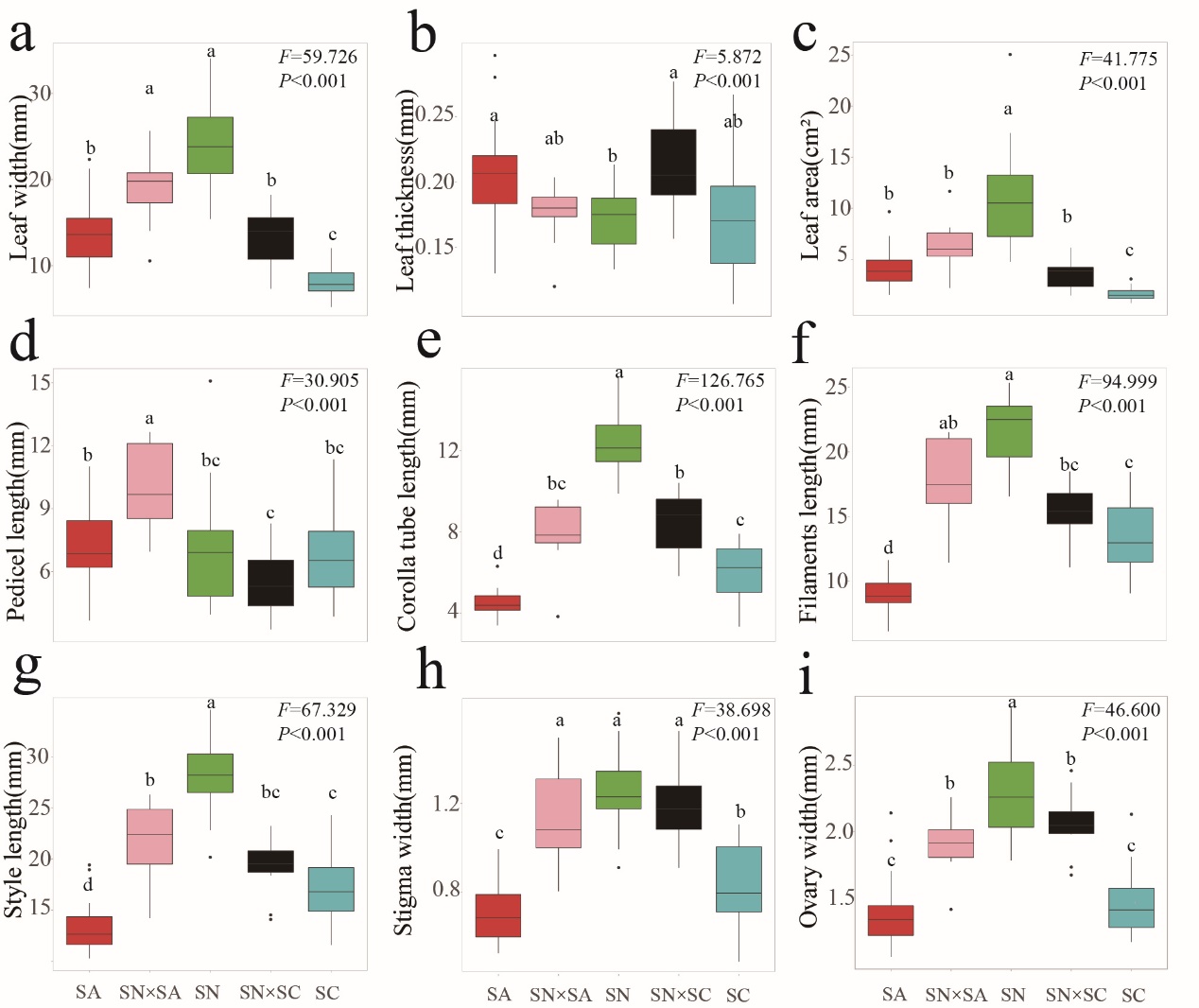


Fig S1. Boxplots of nine quantitative traits for parental species and hybrids of *Rhododendron*. a. leaf width; b. leaf thickness; c. leaf area; d. pedicel length; e. corolla tube length; f. filaments length; g. style length; h. stigma width; and i. ovary width. (*R. spiciferum* [SC], *R. spinuliferum* [SN], *R. scabrifolium* [SA], *R. ×duclouxii* [SN×SC], and the novel hybrid [SN×SA])


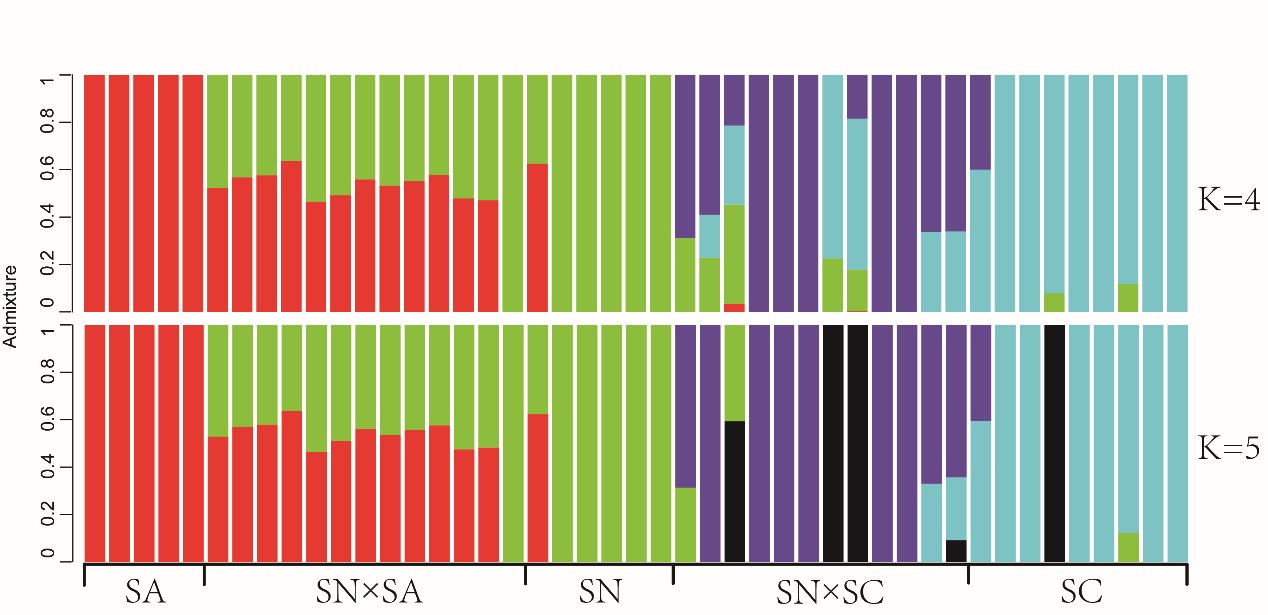


Fig S2. The Admixture result (K=4 & K=5) of the parental species of *Rhododendron* and their hybrids. (*R. spiciferum* [SC], *R. spinuliferum* [SN], *R. scabrifolium* [SA], *R. ×duclouxii* [SN×SC], and the novel hybrid [SN*×*SA]).
